# Connexin43 mediates NF-κB signalling activation induced by high glucose in GMCs: involvement of c-Src

**DOI:** 10.1186/1478-811X-11-38

**Published:** 2013-05-29

**Authors:** Xi Xie, Tian Lan, Xiuting Chang, Kaipeng Huang, Juan Huang, Shaogui Wang, Cheng Chen, Xiaoyan Shen, Peiqing Liu, Heqing Huang

**Affiliations:** 1 Laboratory of Pharmacology & Toxicology, School of Pharmaceutical Sciences, Sun Yat-sen University, Guangzhou, 510006, China; 2 Department of Pharmaceutical Engineering, Ocean College, Hainan University, Haikou, 570228, China; 3 Vascular Biology Institute, Guangdong Pharmaceutical University, Guangzhou, 510006, China

**Keywords:** Connexin43, NF-κB signalling, c-Src, Diabetic nephropathy, Inflammation, Fibronectin

## Abstract

**Background:**

Nuclear factor kappa-B (NF-κB) signalling plays an important role in diabetic nephropathy. Altered expression of connexin43 (Cx43) has been found in kidneys of diabetic animals. The aim of the current study was to investigate the role of Cx43 in the activation of NF-κB induced by high glucose in glomerular mesangial cells (GMCs) and to determine whether c-Src is involved in this process.

**Results:**

We found that downregulation of Cx43 expression induced by high glucose activated NF-κB in GMCs. Orverexpression of Cx43 attenuated NF-κB p65 nuclear translocation induced by high glucose. High glucose inhibited the interaction between Cx43 and c-Src, and enhanced the interaction between c-Src and IκB-α. PP2, a c-Src inhibitor, also inhibited the tyrosine phosphorylation of IκB-α and NF-κB p65 nuclear translocation induced by high glucose. Furthermore, overexpression of Cx43 or inhibition of c-Src attenuated the upregulation of intercellular adhesion molecule-1 (ICAM-1), transforming growth factor-beta 1 (TGF-β1) and fibronectin (FN) expression induced by high glucose.

**Conclusions:**

In conclusion, downregulation of Cx43 in GMCs induced by high glucose activates c-Src, which in turn promotes interaction between c-Src and IκB-α and contributes to NF-κB activation in GMCs, leading to renal inflammation.

## Background

Diabetic nephropathy (DN) is one of the most serious microvascular complications of diabetes and the leading cause of end-stage renal failure. DN is characterized by excessive deposition of extracellular matrix (ECM) proteins, such as fibronectin (FN) and collagen, in the glomerulus and renal tubulointerstitium [1,2]. Hyperglycemia is the primary pathogenetic factor for diabetic renal diseases. In recent years, inflammation has emerged as a key pathophysiological mechanism of DN. Chronic low-grade inflammation and activation of the innate immune system play significant roles in the pathogenesis of DN [3,4]. In diabetes, activated nuclear factor-κB (NF-κB) translocates into the nucleus and triggers the expression of its target genes, including intercellular adhesion molecule-1 (ICAM-1) and transforming growth factor-beta 1 (TGF-β1). These genes cause persistent and enhanced inflammation, leading to excessive FN production and ECM accumulation. Consequently, the pathogenesis of glomerular sclerosis and tubulointerstitial fibrosis are accelerated [5-7]. However, the mechanisms by which high glucose activates NF-κB in DN remain to be explored.

Gap junctional intercellular communication (GJIC) relies on the presence of intercellular protein channels that span the lipid bilayers of contiguous cells, allowing them to directly exchange ions and small molecules [8]. In vertebrates, gap junctions are comprised of a multi-gene family called connexins, among which connexin43 (Cx43) is expressed the most extensively [9]. Glomerular mesangial cells (GMCs) are highly coupled by Cx43-containing gap junctions. Expression level of Cx43 has been reported to parallel the function of GJIC [10,11]. Several studies have demonstrated that Cx43 is involved in the pathogenesis of DN. For example, protein level of Cx43 has been reported to decrease in the kidneys of diabetic patients and animals. Altered gap junctional communication, including abnormality in Cx43, plays a role in altered renal auto-regulation in diabetes [12,13]. Decreased Cx43 is also found in high glucose-treated GMCs. Downregulation of Cx43 induced by high glucose results in senescence and hypertrophy of GMCs [11,14].

The intracellular carboxy tail of Cx43 (Cx43CT) interacts with numerous signalling and scaffolding proteins and thereby regulates cell functions such as cell adhesion, migration, and proliferation [8,15]. Cx43CT interacts with c-Src, a non-receptor tyrosine kinase that can regulate cell proliferation. Activated c-Src phosphorylates Cx43 on the critical tyrosine residues, Tyr247 and Tyr265, and reduces intercellular communication and Cx43 internalisation [16,17]. High glucose-induced protein kinase C and c-Src-dependent big mitogen-activated protein kinase 1 activation are reportedly involved in the pathogenesis of DN [18]. A recent study has shown that activation of c-Src mediates platelet-derived growth factor-induced smad1 phosphorylation and contributes to the progression of glomerulosclerosis in glomerulonephritis [19].

As mentioned above, decreased Cx43 and activated c-Src, which interacts with Cx43CT, are associated with the pathogenesis of DN. Here, we investigated the role of Cx43 in the activation of NF-κB induced by high glucose in GMCs to determine whether c-Src is involved in this process. In addition, we elucidated the molecular mechanism linking these cellular events.

## Results

### Cx43 expression is downregulated and c-Src activity is enhanced in the kidneys of diabetic animals and GMCs exposed to high glucose

**Figure 1:**
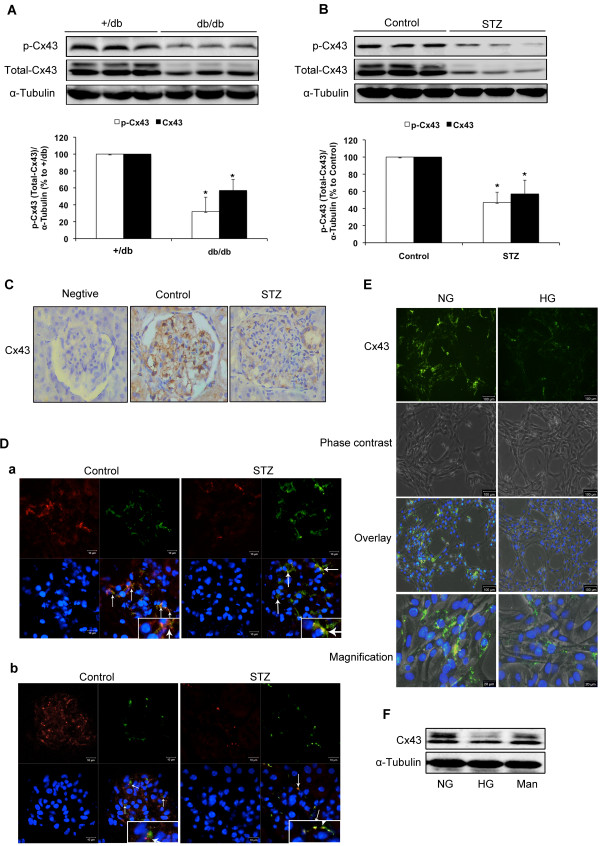
**Cx43 expression is decreased in diabetic kidneys and high glucose-induced GMCs.** (**A, B**) Phosphorylation of Cx43 and total Cx43 expression in kidneys of *db/db* mice and STZ-induced diabetic rats were detected by immunoblotting. (**C**) Cx43 expression was measured by immunohistochemistry in kidneys of STZ-induced rats. Staining without the Cx43 antibody was used as a negative control. (**D**) Images of frozen kidney sections from STZ-induced diabetic rats stained doubly with anti-Cx43 antibody and anti-Thy-1.1 antibody (**a**) or anti-RECA-1 antibody (**b**). Red fluorescence indicates Cx43. Green fluorescence indicates thy-1.1 (**a**) or RECA-1 (**b**). Blue fluorescence indicates nuclei. Scale bar represents 10 μm (magnification 400×). (**E**) GMCs were cultured in DMEM containing normal glucose (NG; 5.5 mmol/L) and serum starved for 16 h before exposure to high glucose (HG; 30 mmol/L). Cx43 expression was measured by immunofluorescence after 30 min of HG stimulation (upper panel). Phase contrast views are also shown (second panel). Green fluorescence indicates Cx43. Scale bar represents 100 μm (magnification 100×). Scale bar represents 20 μm in magnification views (lower panel, magnification 400×). (**F**) Cx43 was measured by immunoblotting after treatment for 30 min with high glucose (30 mmol/L). Mannitol (30 mmol/L) was used as an osmotic control. Experiments were performed at least three times with similar results. ^*^P<0.05 vs. control group.

**Figure 2:**
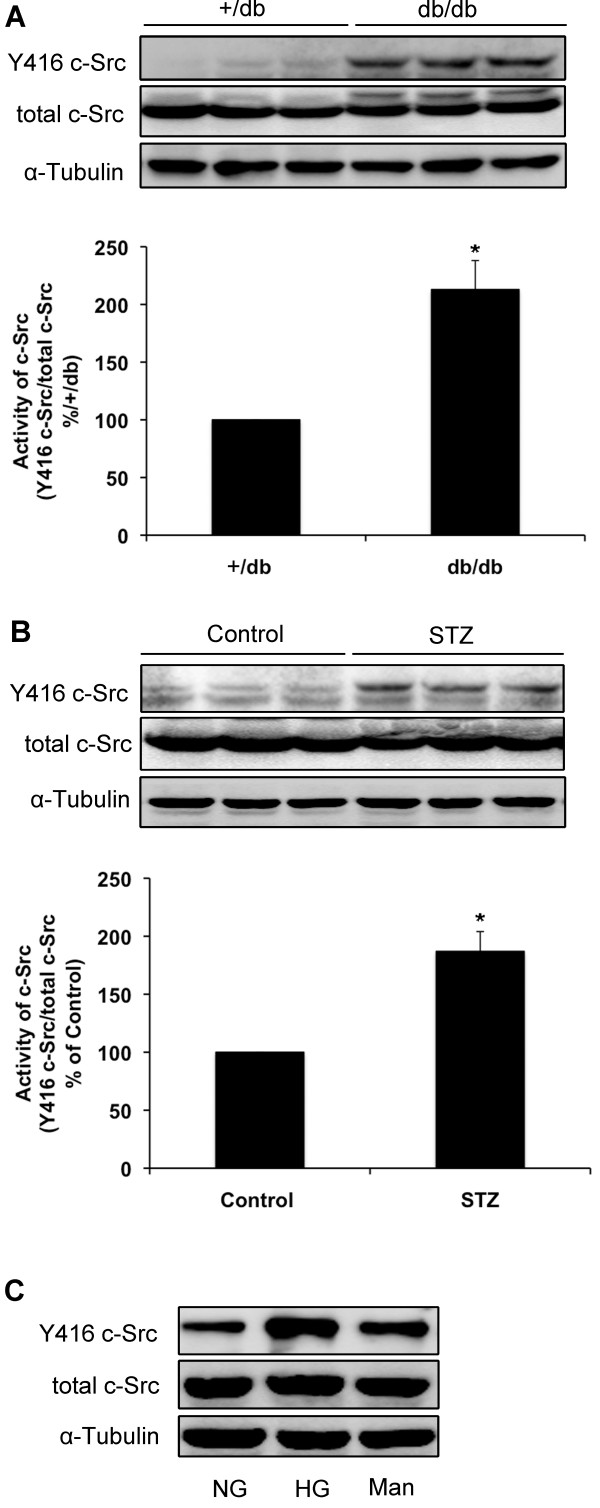
**c-Src activity is upregulated in diabetic kidneys and high glucose-induced GMCs.** (**A, B**) c-Src activity in kidneys of *db/db* mice and STZ-induced diabetic rats was detected by immunoblotting. (**C**) c-Src activity in GMCs was measured by immunoblotting for phosphorylation of Tyr416 on c-Src after treatment for 30 min with high glucose (30 mmol/L) and reprobed with an anti-c-Src antibody as a loading control. Mannitol (30 mmol/L) was used as an osmotic control. Experiments were performed at least three times with similar results. ^*^P<0.05 vs. control group.

We examined expression of Cx43 in diabetic kidneys of diabetic (*db/db*) mice and STZ-induced diabetic rats by immunoblotting. Compared with normal animals, phosphorylated form of Cx43 and total Cx43 protein levels were reduced in the kidneys of both diabetic animals (*P*<0.05; Figures 1A and B). Immunohistological staining also showed lower positive expression of Cx43 in the kidneys of STZ-induced diabetic rats compared with normal rats (Figure 1C). Double immunolabeling of frozen kidney sections showed that Cx43 is expressed in both mesangial and endothelial cells. Furthermore, downregulation of Cx43 was observed in both cell types in the kidneys of STZ-induced diabetic rats (Figure 1D). High glucose (30 mmol/L) treatment for 30 min decreased Cx43 expression in GMCs, whereas mannitol (30 mmol/L) treatment for the same duration exhibited no such effect (Figure 1F). Immunofluorescence results confirmed the decrease in Cx43 expression in GMCs cultured in 30 mM glucose (Figure 1E). c-Src Y416 phosphorylation was found to be upregulated in the kidneys of *db/db* mice and STZ-induced diabetic rats, and the total amount of c-Src remained constant throughout the experiment (*P*<0.05; Figures 2A and B). In addition, high glucose induced significant increase in c-Src Y416 phosphorylation in GMCs but not in the total amount of c-Src (Figure 2C).

### Cx43 and c-Src are responsible for NF-κB activation induced by high glucose in GMCs

**Figure 3:**
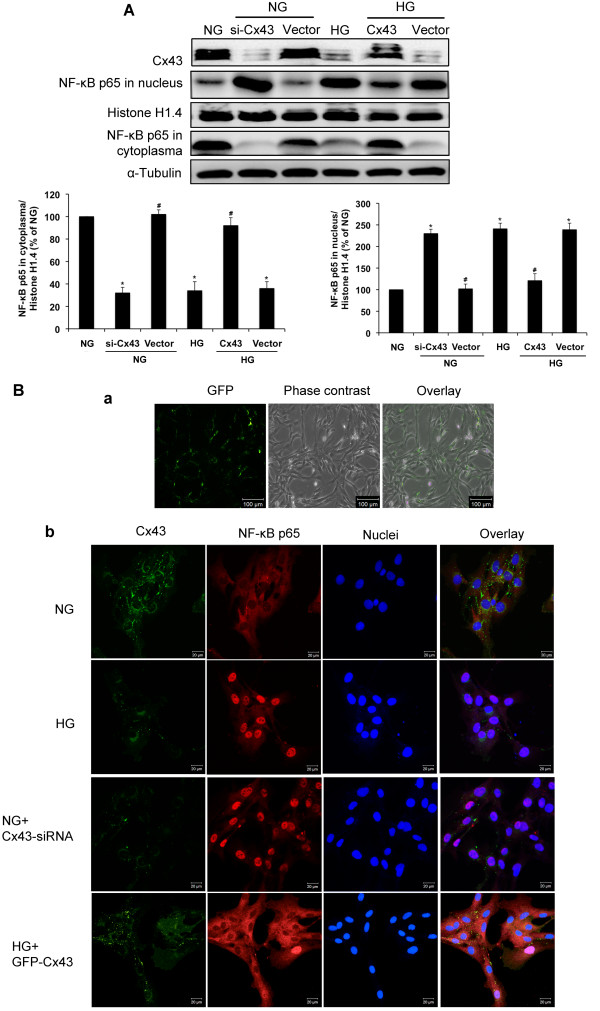
**Cx43 regulates high glucose-induced NF-κB nuclear translocation.** (**A**) GMCs were transfected with Cx43-siRNA or GFP-Cx43 in normal glucose (NG; 5.5 mmol/L). After 48 h of transfection, GMCs were exposed to high glucose (HG; 30 mmol/L) for 30 min. Proteins were then extracted for analysis of Cx43 expression and NF-κB p65 nuclear translocation by immunoblotting. (**B**) Immunofluorescence images of GFP were captured under a laser scanning confocal microscope to assess the transfection efficiency of GFP-Cx43. Green fluorescence indicates GFP. Scale bar represents 100 μm (magnification 100×, **a**). Immunofluorescence images stained doubly with anti-Cx43 antibody and anti-NF-κB p65 antibody were captured under a laser scanning confocal microscope (magnification 400×, **b**). High glucose and Cx43-siRNA-transfection enhance NF-κB p65 nuclear translocation and GFP-Cx43-transfection inhibits high glucose-induced NF-κB p65 nuclear translocation. Red fluorescence indicates localization of NF-κB p65. Green fluorescence indicates localization of Cx43. Blue fluorescence indicates nuclei. Scale bar represents 20 μm. Experiments were performed at least three times with similar results. ^*^P<0.05 vs. normal glucose-treated group, ^#^*P*<0.05 vs. 30 mmol/L glucose-treated group.

**Figure 4:**
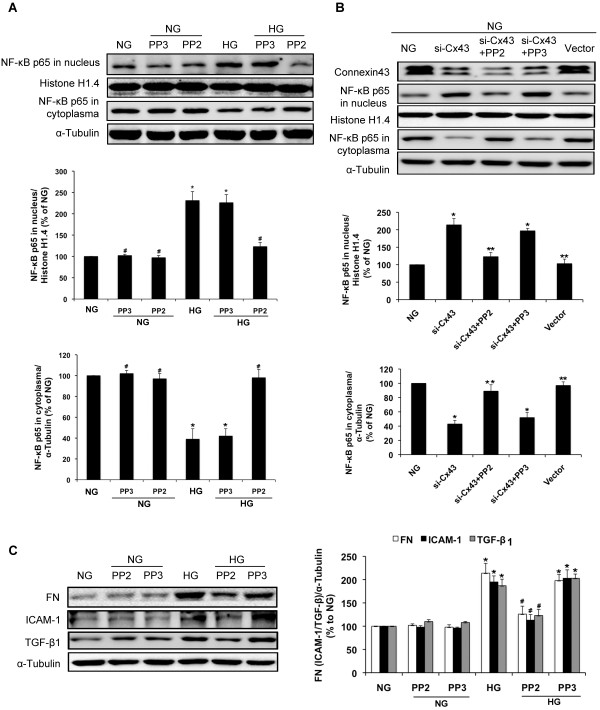
**c-Src regulates high glucose or Cx43-induced NF-κB p65 nuclear translocation.** (**A**) GMCs were preincubated and maintained in 10 μM PP2 (c-Src inhibitor) or 10 μM PP3 (inactive analogue) for 30 min and until the end of the experiment. Cells were then incubated in normal glucose (NG; 5.5 mmol/L) or high glucose (HG; 30 mmol/L) for 30 min. Proteins were extracted for analysis of NF-κB p65 nuclear translocation by immunoblotting. (**B**) GMCs were transfected with Cx43-siRNA in normal glucose (NG; 5.5 mmol/L). After 48 h of transfection, GMCs were co-incubated with PP2 or PP3 (10 μM) for 30 min. Proteins were then extracted for analysis of Cx43 expression and NF-κB p65 nuclear translocation by immunoblotting. (**C**) GMCs were co-incubated with 10 μM PP2 (c-Src inhibitor) or 10 μM PP3, maintained in high glucose for 24 h and then the proteins were extracted for analysis of FN, ICAM-1, and TGF-β1 by immunoblotting. Experiments were performed at least three times with similar results. ^*^P<0.05 vs. normal glucose-treated group, ^#^*P*<0.05 vs. 30 mmol/L glucose-treated group. ^**^P<0.05 vs. Cx43-siRNA transfected group.

Cx43 is known to be regulated by NF-κB [20,21]. Therefore, we sought to determine whether NF-κB is regulated by Cx43 in GMCs exposed to high glucose. We transfected GMCs with plasmids expressing Cx43-siRNA and GFP-Cx43, and analyzed Cx43 expression by immunoblotting. Our results showed that Cx43 expression was decreased by about 70% after Cx43-siRNA transfection, but increased by about 80% after GFP-Cx43 transfection. The empty vector had no effect on Cx43 expression. Immunofluorescence images of GFP-Cx43-transfected cells are shown in Figure 3B (a). Interestingly, nuclear translocation of NF-κB p65 by high glucose and Cx43-silencing was maintained in normal glucose. Furthermore, overexpression of Cx43 using GFP-Cx43 plasmid decreased NF-κB p65 activity in the nuclei of GMCs cultured in high glucose (*P*<0.05; Figure 3A). Immunofluorescence images also showed that high glucose and Cx43-siRNA transfection enhanced NF-κB p65 nuclear translocation while GFP-Cx43-transfection inhibited high glucose-induced NF-κB p65 nuclear translocation (Figure 3B (b)).

c-Src is reportedly involved in NF-κB activation [22,23]. In a previous study, we showed that high glucose induces nucleus translocation NF-κB p65 [24]. In the current study, we found that preincubation with PP2 (10 μM), an inhibitor of c-Src, prevented the increase in NF-κB p65 in the nuclei induced by high glucose (*P*<0.05; Figure 4A). Furthermore, PP2 also prevented nuclear translocation of NF-κB induced by Cx43 siRNA, suggesting the important role of c-Src in NF-κB activation induced by Cx43 (*P*<0.05; Figure 4B). PP2 also inhibited the upregulation of ICAM-1, TGF-β1, and FN expression induced by high glucose in GMCs (*P*<0.05; Figure 4C). An inactive analogue PP3 was used as a control and showed no effect.

### High glucose induces dissociation between Cx43 and c-Src and enhances interaction between c-Src and IκB-α in GMCs

**Figure 5:**
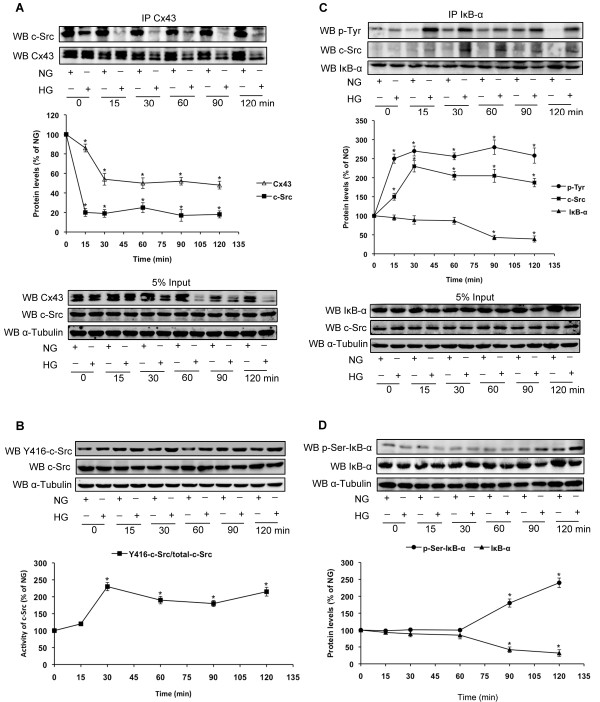
**High glucose induces dissociation of Cx43 and c-Src, and promotes interaction between c-Src and IκB-α.** GMCs were incubated in normal glucose (NG; 5.5 mmol/L) or high glucose (HG; 30 mmol/L) for the indicated times. (**A**) Cx43 was immunoprecipitated with an anti-Cx43 antibody and c-Src was analyzed by immunoblotting. white triangles=Cx43; black squares=c-Src. (**B**) Phosphorylation of tyr416 on c-Src (Y416-c-Src) and total c-Src were analyzed by immunoblotting. α-Tubulin was measured by immunoblotting as a loading control. (**C**) IκB-α was immunoprecipitated with an anti-IκB-α antibody and Tyr-phosphorylation of IκB-α and c-Src were analyzed by immunoblotting. black circle= p-Tyr; black squares=c-Src; black triangles=IκB-α. (**D**) Ser-phosphorylation of IκB-α and total IκB-α were analyzed by immunoblotting. black circle=p-Ser-IκB-α; black triangles=IκB-α. α-Tubulin was measured by immunoblotting as a loading control. Experiments were performed at least three times with similar results. ^*^P<0.05 vs. normal glucose-treated group.

Given the observations above, we further investigated the molecular mechanisms by which Cx43 mediates NF-κB signalling in GMCs exposed to high glucose. The relationships among Cx43, c-Src and IκB-α were investigated by co-immunoprecipitation and immunoblotting. Co-immunoprecipitation results revealed that high glucose decreased Cx43 and induced dissociation between Cx43 and c-Src (*P*<0.05; Figure 5A). Y416 c-Src expression was also increased without changes in the total amount of c-Src by high glucose (*P*<0.05; Figure 5B). Furthermore, direct interaction between c-Src and IκB-α and tyrosine phosphorylation of IκB-α were observed (*P*<0.05; Figure 5C). All of these changes were observed at 15 min of high glucose treatment and persisted for at least 120 min. Serine phosphorylation of IκB-α and degradation of IκB-α were also observed by immunoblotting at 90 min of high glucose treatment, later than the emergence of NF-κB p65 nuclear translocation (*P*<0.05; Figure 5D).

### Immunofluorescence images show the locations of Cx43, c-Src, and IκB-α in GMCs

**Figure 6:**
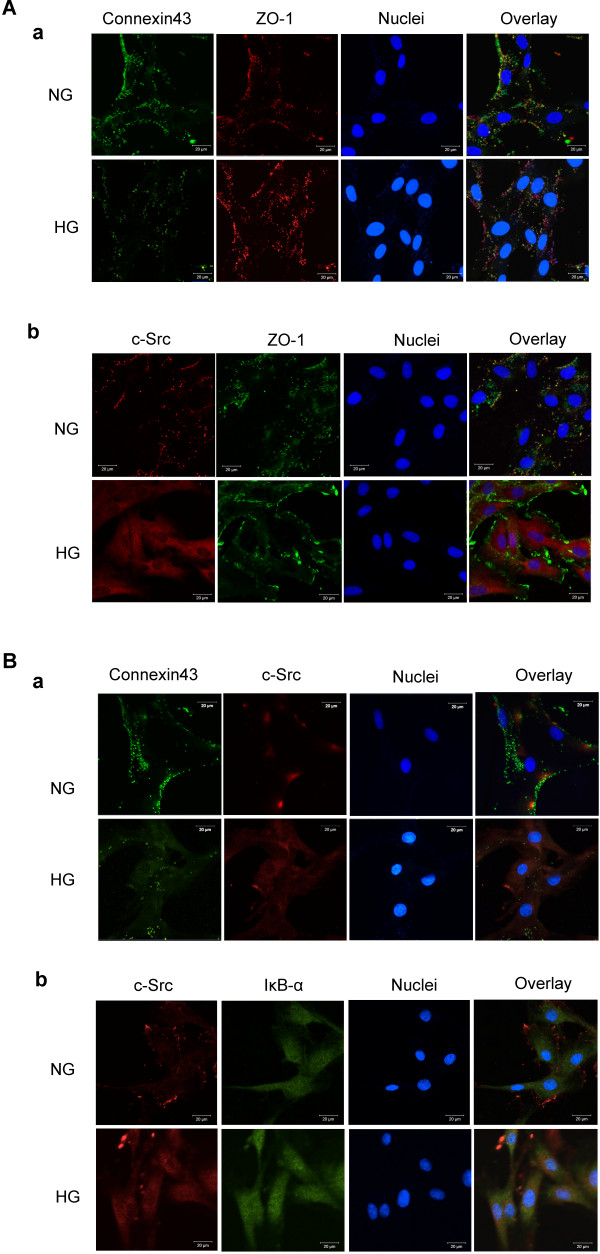
**Immunofluorescence images of co-localization of Cx43, c-Src and IκB-α in GMCs.** GMCs were incubated in normal glucose (NG; 5.5 mmol/L) or high glucose (HG; 30 mmol/L) for 30 min. (**A** (**a**)) Confocal microscopy was used to evaluate the localization of Cx43 under normal glucose or high glucose conditions. Green fluorescence indicates localization of Cx43. Red fluorescence indicates ZO-1, which served as a cytomembrane marker. Blue fluorescence indicates nuclei. (**A** (**b**)) Confocal microscopy was used to evaluate the localization of c-Src under normal glucose or high glucose conditions. Red fluorescence indicates c-Src. Green fluorescence indicates localization of ZO-1. Blue fluorescence indicates nuclei. (**B** (**a**)) Confocal microscopy was used to evaluate the localization of Cx43 and c-Src under normal glucose or high glucose conditions. A significant decrease in Cx43 was observed after high glucose treatment. c-Src dissociated from Cx43 and translocated into the cytoplasm after high glucose treatment. Green fluorescence indicates localization of Cx43. Red fluorescence indicates c-Src localization. Blue fluorescence indicates nuclei. (**B** (**b**)) Confocal microscopy was used to evaluate the localization of c-Src and IκB-α. c-Src was translocated into the cytoplasm from the cytomembrane after high glucose treatment and then co-localized with IκB-α in the cytoplasm of GMCs. Green fluorescence indicates localization of IκB-α. Red fluorescence indicates c-Src localization. Blue fluorescence indicates nuclei. Scale bar represents 20 μm (magnification 630×).

We next performed immuofluorescence staining of Cx43, c-Src, and IκB-α in GMCs to confirm our co-immunoprecipitation results. Zonula occludens-1 (ZO-1), originally identified as a component of tight junctions, is a member of the membrane-associated guanylate kinase family of proteins that interacts with Cx43 at the plasma membrane [25]. Cx43 and ZO-1 were found to co-localize at the membrane of GMCs cultured in normal glucose. However, a significant decrease in the membrane Cx43 of GMCs was observed after 30 min of high glucose treatment (Figure 6A (a)). c-Src was also found to be located on the membrane of GMCs cultured in normal glucose. High glucose induced its translocation to the cytoplasm (Figure 6A (b)). Furthermore, an abundance of Cx43 and the majority of c-Src were found to localize on the membrane of GMCs maintained in normal glucose (Figure 6B (a)). No co-localization was observed between c-Src and IκB-α (Figure 6B (b)). However, Cx43 expression on the membrane decreased after high glucose treatment (Figure 6B (a).) Meanwhile, c-Src was translocated to the cytoplasm of GMCs, where it interacted with IκB-α (Figure 6B (b)).

### Cx43 and c-Src regulate tyrosine phosphorylation of IκB-α induced by high glucose in GMCs

**Figure 7:**
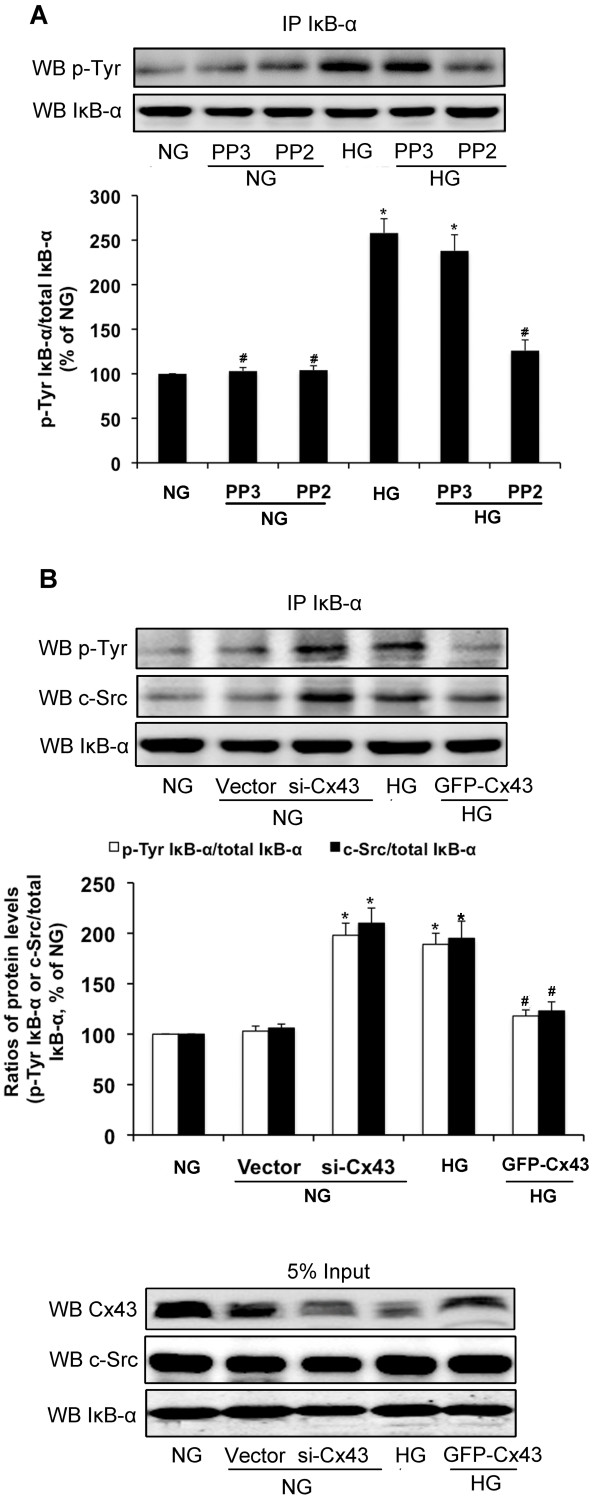
**Cx43 and c-Src are responsible for high glucose-induced Tyr-phosphorylation of IκB-α.** (**A**) GMCs were preincubated and maintained in 10 μM PP2 (c-Src inhibitor) or 10 μM PP3 (inactive analogue of PP2) for 30 min and until the end of the experiment. Cells were then incubated in normal glucose (NG; 5.5 mmol/L) or high glucose (HG; 30 mmol/L) for 30 min and IκB-α was immunoprecipitated with an anti-IκB-α antibody. Tyr-phosphorylation of IκB-α and total-IκB-α were then analyzed by immunoblotting. (**B**) GMCs were transfected with Cx43-siRNA or GFP-Cx43 under the condition of normal glucose (NG; 5.5 mmol/L). After 48 h of transfection, GMCs were exposed to high glucose (HG; 30 mmol/L) for 30 min. IκB-α was immunoprecipitated with an anti-IκB-α antibody, and Tyr-phosphorylation of IκB-α and c-Src, and total-IκB-α were analyzed by immunoblotting. Experiments were performed at least three times with similar results. ^*^P<0.05 vs. normal glucose-treated group, ^#^*P*<0.05 vs. 30 mmol/L glucose-treated group.

To determine whether regulation of NF-κB by Cx43 and c-Src involves tyrosine phosphorylation of IκB-α, plasmids of Cx43-siRNA and GFP-Cx43, and PP2, a c-Src inhibitor, were used. High glucose alone and transfection of Cx43-siRNA induced tyrosine phosphorylation of IκB-α and interaction between c-Src and IκB-α in GMCs cultured in normal glucose. However, pretreatment with PP2 (10 μM) significantly inhibited tyrosine phosphorylation of IκB-α induced by high glucose (*P*<0.05; Figure 7A). Restoration of Cx43 by transfection of GFP-Cx43 decreased tyrosine phosphorylation of IκB-α and interaction between c-Src and IκB-α induced by high glucose (*P*<0.05; Figure 7B).

### Cx43CT plays an important role in the regulation of NF-κB by Cx43 independent of GJIC

**Figure 8:**
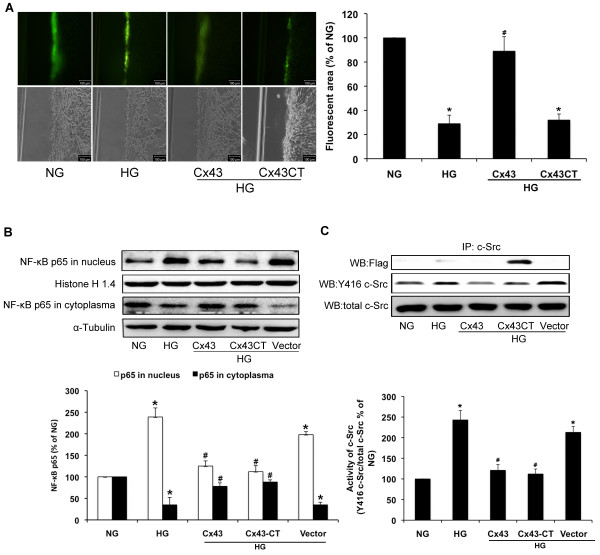
**Regulation of high glucose-induced NF-κB nuclear translocation and c-Src activity by Cx43 is independent of GJIC.** GMCs were transfected with GFP-Cx43 or Flag-Cx43CT under the condition of normal glucose (NG; 5.5 mmol/L). After 48 h of transfection, GMCs were exposed to high glucose (HG; 30mmol/L) for 30 min. (**A**) Photomicrographs obtained after Lucifer yellow scrape-loading in GMCs transfected with GFP-Cx43 and Flag-Cx43CT (magnification 100×, upper panel). Phase contrast views are also provided (lower panel). Scale bar represents 100 μm. (**B**) Proteins were extracted for analysis of NF-κB p65 nuclear translocation by immunoblotting. Histone H1.4 and α-tubulin were measured by immunoblotting as a loading control. (**C**) c-Src was immunoprecipitated with an anti-c-Src antibody and Flag, phosphorylation of Tyr416 on c-Src (Y416-c-Src) and total c-Src were analyzed by immunoblotting. Experiments were performed at least three times with similar results. ^*^P<0.05 vs. normal glucose-treated group, ^#^*P*<0.05 vs. 30 mmol/L glucose-treated group.

Flag-Cx43CT, which consists of the intracellular carboxy tail of Cx43 tagged with FLAG, was used to determine whether regulation of NF-κB by Cx43 is independent of GJIC. Results of scrape-loading experiments showed that GJIC inhibited by high glucose was restored by transfection of GFP-Cx43. Flag-Cx43CT did not show any effect (Figure 8A). Like GFP-Cx43, transfection with Flag-Cx43CT also significantly inhibited high glucose-induced NF-κB p65 nuclear translocation (*P*<0.05; Figures 8B). Additionally, transfection with Flag-Cx43CT exhibited an inhibitory effect on c-Src activation induced by high glucose in GMCs. Our observation of co-immunoprecipitation between c-Src and FLAG suggests a direct interaction between Flag-Cx43CT and c-Src (*P*<0.05; Figures 8C).

### Cx43 inhibits upregulation of ICAM-1, TGF-β1, and FN expression induced by high glucose in GMCs

**Figure 9:**
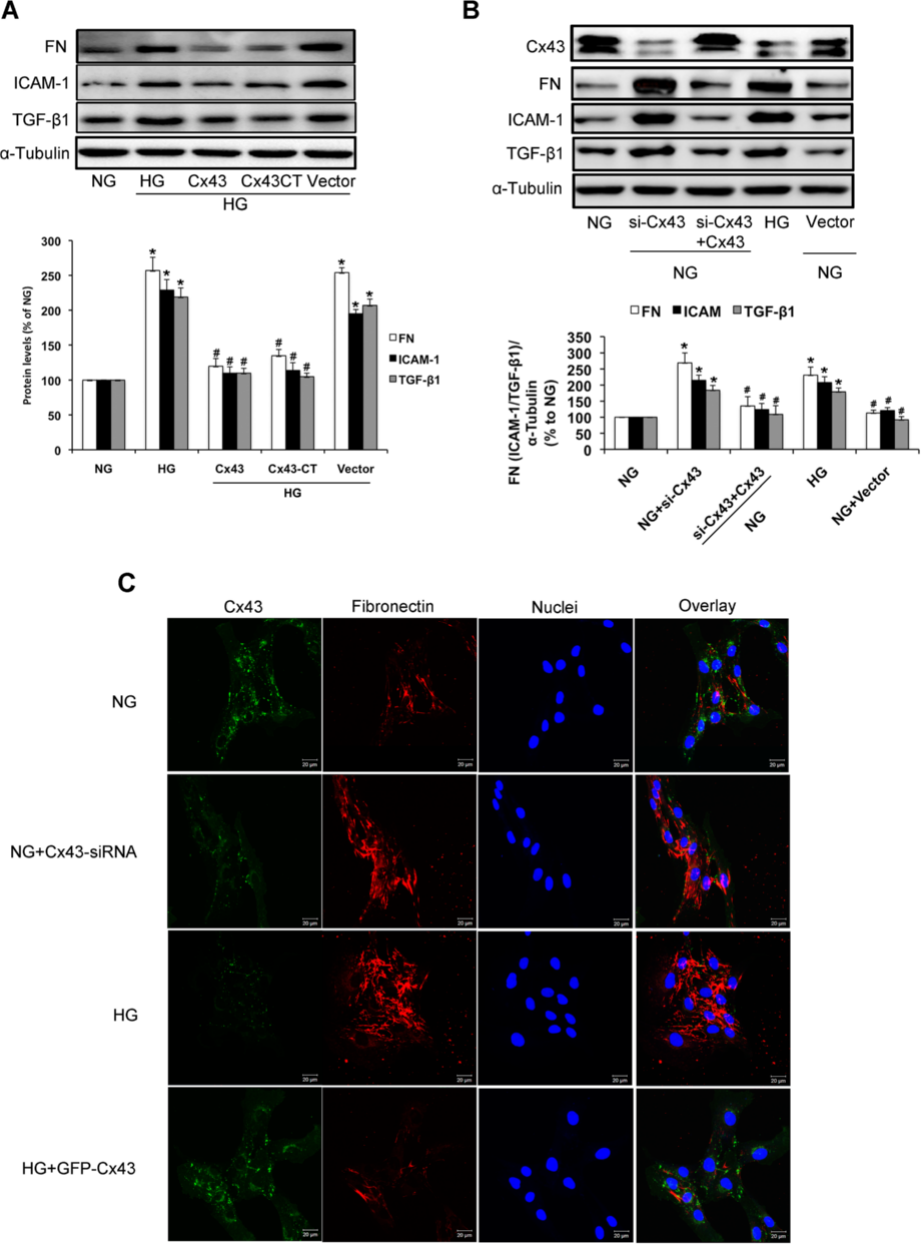
**Cx43 inhibits high glucose-induced expression of FN, ICAM-1, and TGF-β1.** (**A**) GMCs were transfected with GFP-Cx43 or Flag-Cx43CT in normal glucose (NG; 5.5 mmol/L). After 48 h of transfection, GMCs were exposed to high glucose (HG; 30 mmol/L) for 24 h. Proteins were extracted for analysis of FN, ICAM-1, and TGF-β1 by immunoblotting. (**B**) GMCs were transfected with Cx43-siRNA in normal glucose. After 24 h, cells were then transfected with GFP-Cx43 to restore Cx43 expression. Proteins were extracted for analysis of FN, ICAM-1, and TGF-β1 by immunoblotting. The high glucose-treated group was used as the control. (**C**) Immunofluorescence images stained doubly with anti-Cx43 antibody and anti-FN antibody were captured under a laser scanning confocal microscope (magnification 630×). Green fluorescence indicates Cx43. Red fluorescence indicates FN. Blue fluorescence indicates nuclei. Scale bar represents 20 μm. Experiments were performed at least three times with similar results. ^*^P<0.05 vs. normal glucose-treated group, ^#^*P*<0.05 vs. 30 mmol/L glucose-treated group.

ICAM-1 and TGF-β1 are well-known important inflammatory factors in the pathogenesis of DN [26-30]. FN is an important ECM component in the kidney [1,2]. Treatment with high glucose for 24 h markedly increased ICAM-1, TGF-β1, and FN protein levels compared with the control group (*P*<0.05; Figure 9A). However, GFP-Cx43 or Flag-Cx43CT-transfection in high glucose-treated GMCs significantly inhibited upregulation of these proteins. Transfection with the vector alone had no effect on the production of ICAM-1, TGF-β1, and FN proteins (*P*<0.05; Figure 9A). Cx43-siRNA had similar effects as high glucose for FN, ICAM-1 and TGF-β1. Restoration of Cx43 by transfection of GFP-Cx43 attenuated FN, ICAM-1 and TGF-β1 accumulation (*P*<0.05; Figure 9B). Immunofluorescence staining also revealed that FN was upregulated by high glucose or Cx43-siRNA, and restoration of Cx43 by GPF-Cx43-transfection attenuated FN upregulation induced by high glucose (Figure 9C).

## Discussion

Downregulation of Cx43 protein expression has been observed in the kidneys of diabetic animals and high glucose-treated GMCs [11-14]. Consistent with previous studies, we observed that the protein level of Cx43 was reduced in the kidneys of *db/db* mice and STZ-induced diabetic rats. Furthermore, significantly reduced Cx43 protein level was observed after 30 min of high glucose exposure in GMCs. Previous studies have reported that the half-life of Cx43 is short- as litter as 1–2 hours [31-33]. We explored the half-life of Cx43 in GMCs cultured in normal glucose or high glucose using cycloheximide. A significant decrease in Cx43 was observed after 30 min of normal glucose (5.5 mM) exposure. However, high glucose (30 mM) induced a faster decrease in Cx43 after 15 min stimulation, suggesting Cx43 is actively degraded (Additional file 1: Figure S1). In our previous study, we found that NF-κB signalling is activated in the kidneys of diabetic rats and high glucose-treated GMCs [24]. While several studies have investigated the relationship between Cx43 and NF-κB signalling, most of them have focused only on the regulation of Cx43 by NF-κB. For instance, AngII has been found to induce binding of NF-κB to the Cx43 gene promoter, increasing Cx43 expression in aortic smooth muscle cells while the TLR3 ligand polyI:C has been observed to induce downregulation of Cx43 by a mechanism involving NF-κB [20,21].

In the present study, we found that downregulation of Cx43 induced by high glucose or transfection with the Cx43-siRNA plasmid enhanced nuclear translocation of NF-κB p65. However, restoration of Cx43 expression by transfection with GFP-Cx43 attenuated high glucose-induced NF-κB p65 nuclear translocation in GMCs, which suggests that decreased Cx43 expression mediates NF-κB activation in GMCs. Thus, our findings show that Cx43 participates in the activation of NF-κB in high glucose-treated GMCs and enhances the relationship between NF-κB and Cx43. The molecular mechanism of this cellular event, however, remains unclear.

We also observed upregulation of c-Src activity in the kidneys of *db/db* mice and STZ-induced diabetic rats. Previous studies have shown that high glucose can activate c-Src [34,35]. Consistent with such findings, our results show that c-Src is activated in high glucose-treated GMCs. c-Src has been proposed to be responsible for the pathogenesis of DN. We used PP2, a c-Src inhibitor, to explore whether c-Src is involved in the high glucose-induced activation of NF-κB signalling in GMCs. We found that PP2 inhibited NF-κB p65 nuclear translocation induced by high glucose or Cx43 silencing, suggesting the important role of c-Src in Cx43-induced NF-κB activation.

As mentioned above, both Cx43 and c-Src are involved in the activation of NF-κB in high glucose-treated GMCs. Therefore, we further explored the molecular mechanisms involved in these events. Previous studies have indicated that phosphorylation of Cx43 by c-Src reduces gap junctional communication depending on the interaction between Cx43CT and c-Src [17,36]. Interestingly, recent studies have suggested that the interaction between Cx43 and c-Src reciprocally modulates their activities. The level of Cx43 expression is important in regulating c-Src activity. Upregulation of Cx43 in glioma cells reduces c-Src activity while silencing of Cx43 activates c-Src in astrocytes [37,38]. In our study, reduction of Cx43 protein level induced by high glucose was accompanied by decrease in the amount of c-Src interacting with Cx43, thereby increasing the activity of c-Src in the cytoplasm. This finding indicates that downregulation of Cx43 by high glucose activates c-Src.

The molecular mechanism by which c-Src regulates NF-κB has been suggested to be dependent on the interaction between c-Src and IκB kinase β (IKKβ) or IκB-α. IKKβ is phosphorylated by c-Src, which is involved in TNF-α-induced ICAM-1 expression [22]. Tyrosine phosphorylation of IκB-α activates NF-κB through a redox-regulated and c-Src-dependent mechanism following hypoxia/reoxygenation [23]. In the current study, IκB-α was found to interact with c-Src after exposure of GMCs to high glucose for 15 min, and to be accompanied by tyrosine phosphorylation of IκB-α, persisting for at least 120 min. We have previously shown that NF-κB p65 is translocated into the nucleus after exposure of GMCs to high glucose levels for 30 min [24]. Interestingly, IKK-mediated serine phosphorylation of IκB-α, a classic pathway of NF-κB activation [39], was detected after exposure of GMCs to high glucose levels for 90 min, and this was accompanied by degradation of IκB-α, which occurs after NF-κB p65 nuclear translocation. Thus, tyrosine phosphorylation of IκB-α could possibly play an important role in the initial step of high glucose-induced NF-κB p65 activation. As described in a previous study, tyrosine phosphorylation activates NF-κB without degradation of IκB-α [40]. We did not observe degradation of IκB-α when NF-κB p65 was translocated into the nucleus at early stages of exposure of GMCs to high glucose.

Immunofluorescence images showed that Cx43 and c-Src were co-localized around the cell membrane in GMCs maintained in normal glucose. There was no interaction between c-Src and IκB-α in GMCs cultured in normal glucose. However, co-localization of c-Src and IκB-α was observed in the cytoplasm after exposure of GMCs to high glucose for 30 min. Based on these data, we propose that decrease in Cx43 expression enhances the activity of c-Src by acting as a substrate of the kinase, which promotes interaction between c-Src and IκB-α and leads to NF-κB activation. The results of our study confirm that PP2, an inhibitor of c-Src, can inhibit the tyrosine phosphorylation of IκB-α and translocation of NF-κB p65 into the nucleus, which suggests that c-Src regulates NF-κB by inducing tyrosine phosphorylation of IκB-α.

A recent study has reported that silencing Cx43 activates c-Src, which in turn upregulates HIF-1α leading to the upregualtion of the machinery required to take up glucose in astrocytes [38]. Thus, c-Src is an important factor in the regulation of nuclear transcription factors by Cx43. In this study, we found that high glucose and silencing of Cx43 induced c-Src activation and promoted interaction between c-Src and IκB-α in GMCs cultured in normal glucose. Restoration of Cx43 greatly attenuated these changes in GMCs cultured in high glucose, confirming that the interaction between c-Src and IκB-α is regulated by Cx43. We also explored the relationship of HIF-1α and Cx43 in GMCs. HIF-1α protein level was upregulated by high glucose or reduced Cx43 level in GMCs. Inhibition of c-Src or NF-κB abrogated the increase in HIF-1α protein level induced by high glucose. The increase in HIF-1α protein level was associated with significant accumulation of FN, ICAM-1 and TFG-β1 in GMCs exposed to high glucose, suggesting a potential role of HIF-1α in the pathogenesis of DN. However, further research is needed to define the role of HIF-1α in DN (Additional file 2: Figure S2).

The regulation of NF-κB by reduced Cx43 protein level could be caused by absence of Cx43 function (gap junctional communication) or absence of Cx43 interactions with other proteins, such as c-Src. Restoration of Cx43CT, a non-channel forming region, increases the expression of the intracellular carboxy tail of Cx43 without affecting GJIC [37]. Consistent with previous observations, our results showed that restoration of Cx43 rebuilt GJIC inhibited by high glucose. However, Cx43CT overexpression did not exhibit such effects. Similar to the restoration of Cx43, Cx43CT reduced the activation of c-Src and NF-κB in GMCs exposed to high glucose, which suggests that this effect depends mostly on the interaction between Cx43CT and c-Src.

Our results confirm our hypothesis that Cx43 regulates the activity of c-Src in high glucose-treated GMCs and activates NF-κB. We further investigated the effects of Cx43 on protein expression of target genes of NF-κB, including ICAM-1 and TGF-β1, in high glucose-treated GMCs. ICAM-1 is an important downstream inflammatory factor whose gene contains an NF-κB binding site in the promoter region [41]. ICAM-1 gene deficiency prevents nephropathy in type 2 diabetic db/db mice [26,27]. TGF-β1 is recognised as another important factor in DN pathogenesis by mediating inflammatory responses, which aggravates accumulation of the ECM proteins FN and collagen, and interstitial myofibroblast activation, a critical event in the pathogenesis of interstitial fibrosis [28-30].

In the current study, restoration of Cx43 or Cx43CT reversed high glucose-induced increases in ICAM-1 and TGF-β1 protein expression in GMCs. FN is an important factor in the ECM and excessive synthesis of it contributes to glomerular basement membrane thickening and glomerular sclerosis. Several studies have proposed that Cx43 may play an important role in cardiac and pulmonary fibrosis. Mice lacking Cx40 and endothelial cell Cx43 have lung dysfunction and fibrosis [42]. Reduced Cx43 expression increases fibrosis and pro-arrhythmia in aged and pressure-overloaded mice due to enhanced fibroblast activity [43]. In our study, high glucose or silencing of Cx43 by Cx43-siRNA induced the upregulation of ICAM-1, TGF-β1 and FN. Overexpression of Cx43 and Cx43CT attenuated the increase in FN induced by high glucose in GMCs, confirming the importance of Cx43 in renal fibrosis. The c-Src inhibitor PP2 also exhibited an inhibitory effect on the overexpression of ICAM-1, TGF-β1 and FN induced by high glucose, thus confirming the role of c-Src in the activation of NF-κB.

## Conclusions

Our study describes a novel mechanism of NF-κB activation in high glucose-treated GMCs involving Cx43. In summary, downregulation of Cx43 induced by high glucose activates c-Src, which in turn promotes interaction between c-Src and IκB-α and contributes to NF-κB activation, leading to renal inflammation. The results presented in this study show that Cx43 induces NF-κB activation and fibrosis in GMCs, which is beneficial for the development of new therapies against DN. However, the mechanism by which regulation of Cx43 expression occurs requires further study.

## Methods

### Cell culture and transfection

Rat GMCs were separated from the glomeruli of Sprague–Dawley (SD) rats and identified via a specific assay as previously described [44]. The cultured cells were used at confluence between the 5^th^ and 8^th^ passages. Confluent cells were rendered quiescent by incubation for 24 h in serum-free medium before treating with glucose (5.5 mmol/L as normal glucose and 30 mmol/L as high glucose) or osmotic control (mannitol, 30 mmol/L final concentration) for various times. 10 μM PP2 (c-Src inhibitors) or 10 μM PP3 (inactive analogue) were added before high glucose for 30 min (Sigma-Aldrich, St. Louis, MO). Transfection of GFP-Cx43, Flag-Cx43CT (Addgene, Cambridge, MA) and Cx43-siRNA plasmid (gift from Tao Liang professor, Zhongshan School of Medicine, SYSU, China) were performed per the manufacturer’s instruction for Lipofectamine™ LTX &Plus Reagent (Life Technologies, Carlsbad, CA).

### Immunoprecipitation and immunoblotting

The cell monolayers were lysed in a cell lysis buffer for immunoprecipitation (Beyotime, Jiangsu, China). Immunoprecipitation was performed by incubating 0.5 mg cell lysate protein which was determined by bicinchoninic acid assay (BCA) according to the manufacture’s instructions (Thermo Fisher Scientific, Rockford, IL) with 1μg of corresponding antibody and protein G/A agarose bead (Merk, Darmstadt, Germany) at 4°C overnight. Immunoblotting was performed as previously described [45]. Kidney tissues were lysed, proteins were extracted as previously published [46]. The nuclear and cytoplasmic proteins of GMCs were extracted using a commercially available assay kit (Active Motif, Carlsbad, CA) and the total proteins were extracted as published [46]. The signals were visualized by a GE ImageQuant LAS4000mini, and analyzed using the Quantity One Protein Analysis Software (Bio-Rad Laboratories, Hercules, CA). The antibodies included mouse monoclonal antibodies against connexin43, NF-κB p65, Inhibitor of κB-α (IκB-α), p-Tyr and FN, rabbit polyclonal antibody against c-Src, goat polyclonal anti-body against ICAM-1 and ZO-1 (Santa Cruz Biotechnology, Santa Cruz, CA), rabbit monoclonal antibodies against phospho-c-Src (Tyr), connexin43, phospho-IκB-α (Ser) and TGF-β (Cell Signalling, Danvers, MA), rabbit monoclonal antibodies against Histone H1.4 and α-tubulin (Sigma–Aldrich, St. Louis, MO), mouse monoclonal antibodies against Thy-1.1 and RECA-1 (Abcam, Cambridge, MA).

### Confocal laser scanning fluorescence microscopy (LSCM)

Different groups of adherent cells were washed with phosphate-buffered saline (PBS), fixed with 4% paraformaldehyde in PBS for 20 min, and permeabilized with 0.1% TritonX-100 for 5 min at room temperature. Cells were incubated with antibodies against NF-κB p65, connexin43, c-Src, IκB-α or FN overnight at 4°C after blocking with 10% goat serum. Or frozen kidney sections (7.5 μm) were incubated with antibodies against Cx43, Thy-1.1 and RECA-1 overnight at 4°C after blocking with 10% goat serum. Then the cells and sections were incubated in the dark at room temperature for 1 h with a secondary antibody (Alexa Fluor® 488, Alexa Fluor® 546, Invitrogen, Carlsbad, CA). The nucleus was stained with Hoechst33342. Cells and sections were placed under a laser scanning confocal microscope (LSM710, Carl Zeiss, Germany) for observation and image acquisition.

### Assessment of gap junctional intercellular communication

Gap junction permeability was determined by the scrape-loading/dye transfer technique [37]. Scrape-loading was performed by scraping the cell layer with a broken razor blade in culture media containing Lucifer yellow (1 mg/ml, Life Technologies, Carlsbad, CA). Lucifer yellow is a low molecular weight (457 Da) fluorescent dye that can pass through the gap junctions of loaded cells to their neighbors. After 2 min, the dye solution was removed and the cells were carefully washed. Subsequently, 5 min after scraping, fluorescence photomicrographs were captured with a laser scanning confocal microscope (LSM710, Carl Zeiss, Germany). At least six photomicrographs of the centre of the dish were taken and the fluorescent area occupied by Lucifer yellow in the images was measured with the image-analyzer software (Scion Image, Scion Corporation, Frederick, MD).

### Animal experiment

Male SD rats (*n*=20, 200±10 g) were obtained from the Laboratory Animal Center, Sun Yat-sen University, Guangzhou, China Animal (Quality Certificate No.: 0005201). *db/db* (male, *n*=10, 40±5 g) mice were obtained from the model animal research center of Nanjing University (Quality Certificate No.: 0007963). All animal procedures conformed to the China Animal Welfare Legislation and were reviewed and approved by the Sun Yat-sen University Committee on Ethics in the Care and Use of Laboratory Animals. All animals were housed under standard conditions with free access to regular food and water. After feeding with regular diet for 1 week, STZ-diabetic rats were induced as previously reported [24]. Diabetic rats were confirmed by the levels of fasting blood glucose measurement (≥16.7 mmol/l after 72 h injection). It was continued for 12 weeks, after which the rats were sacrificed. *db/db* mice were sacrificed at the time when they were 12 weeks age. Kidney samples were rapidly excised, weighed and frozen in liquid nitrogen and then stored at -80°C or fixed in 10% neutral-buffered formalin.

### Immunohistochemistry

Kidney sections 4 μm thick were processed using a standard immunostaining protocol as previously reported [47]. A negative control was prepared by omitting the primary antibody.

### Statistical analysis

All experiments were performed at least in triplicate. The data were assessed using SPSS 11.5. All values were expressed as mean ± SD. Statistical analyses of data were performed by one-way ANOVA using post-hoc multiple comparisons. *P* < 0.05 was considered to be statistically significant.

## Abbreviations

Cx43: Connexin43; Cx43CT: Carboxy tail of Cx43; DN: Diabetic nephropathy; ECM: Extracellular matrix; FN: Fibronectin; GJIC: Gap junctional intercellular communication; GMCs: Glomerular mesangial cells; ICAM-1: Intercellular adhesionmolecule-1; IKK: IκB kinase; IκB-α: Inhibitor of κB-α; NF-κB: Nuclear factor kappa-B; TGF-β1: Transforming growth factor-beta 1; SD rats: Sprague–Dawley rats.

## Competing interest

The authors declare that there is no conflict of interest associated with this manuscript.

## Authors’ contributions

XX designed and performed experiments, acquisition and analysis of data, and drafted the manuscript. TL, XTC and KPH helped to perform experiments and prepare the manuscript. JH, SGW and CC have conceived of the study, participated in its design and coordination. HQH, PQL and XYS have been involved in drafting the manuscript and revising it critically for important intellectual content. All authors have read and approved the final version of this manuscript.

## Supplementary Material

Additional file 1: Figure S1 Half-life of Cx43 was explored in GMCs cultured in normal glucose or high glucose using cycloheximide. (**A**) GMCs cultured in normal glucose (5.5 mM) were co-incubated with 10 μM cycloheximide for the indicated time. (**B**) GMCs cultured in high glucose (30 mM) were co-incubated with 10 μM cycloheximide for the indicated time. (**C**) GMCs were treated for 30 min with increasing concentrations of glucose as indicated. Then proteins were extracted for analysis of Cx43 by immunoblotting. α-Tubulin was used as a loading control. ^*^P<0.05 vs. control group. Chx, cycloheximide. Click here for file

Additional file 2: Figure S2 HIF-1α is regulated in the GMCs by high glucose or low levels of Cx43. (**A** and **B**) GMCs were treated with high glucose for the indicated time, then proteins were extracted for analysis of HIF-1α by immunoblotting. (**C**) GMCs were transfected with Cx43-siRNA in normal glucose (5.5 mM) or GFP-Cx43 in high glucose (30 mM). After 48 h, proteins were extracted for analysis of Cx43 and HIF-1α by immunoblotting. (**D**) GMCs cultured in high glucose were co-incubated with PP2 or PP3 (10 μM) or PDTC (100 μM). After 48 h, proteins were extracted for analysis of HIF-1α by immunoblotting. α-Tubulin was used as a loading control. ^*^P<0.05 vs. normal glucose-treated group. ^#^*P*<0.05 vs. high glucose-treated group. Click here for file
