# Supplementary figures and images for: Connexin43 mediates NF-κB signalling activation induced by high glucose in GMCs: involvement of c-Src
Source: Cell Commun Signal. 2013 May 29;11:38. doi: 10.1186/1478-811X-11-38 (PMC3699363; doi:10.1186/1478-811X-11-38)

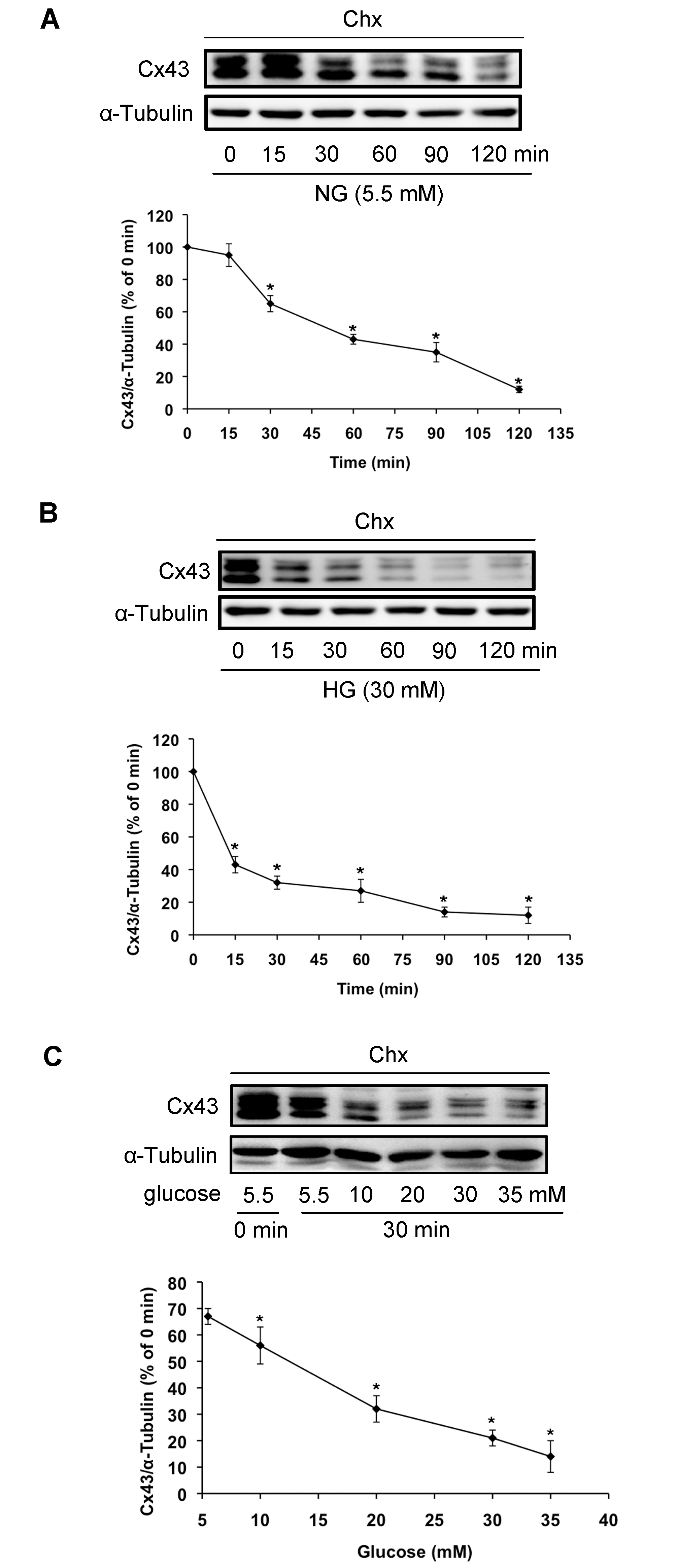

Supplement: Additional file 1: Figure S1 — Half-life of Cx43 was explored in GMCs cultured in normal glucose or high glucose using cycloheximide. (A) GMCs cultured in normal glucose (5.5 mM) were co-incubated with 10 μM cycloheximide for the indicated time. (B) GMCs cultured in high glucose (30 mM) were co-incubated with 10 μM cycloheximide for the indicated time. (C) GMCs were treated for 30 min with increasing concentrations of glucose as indicated. Then proteins were extracted for analysis of Cx43 by immunoblotting. α-Tubulin was used as a loading control. *P<0.05 vs. control group. Chx, cycloheximide. [file 1478-811X-11-38-S1.tiff]

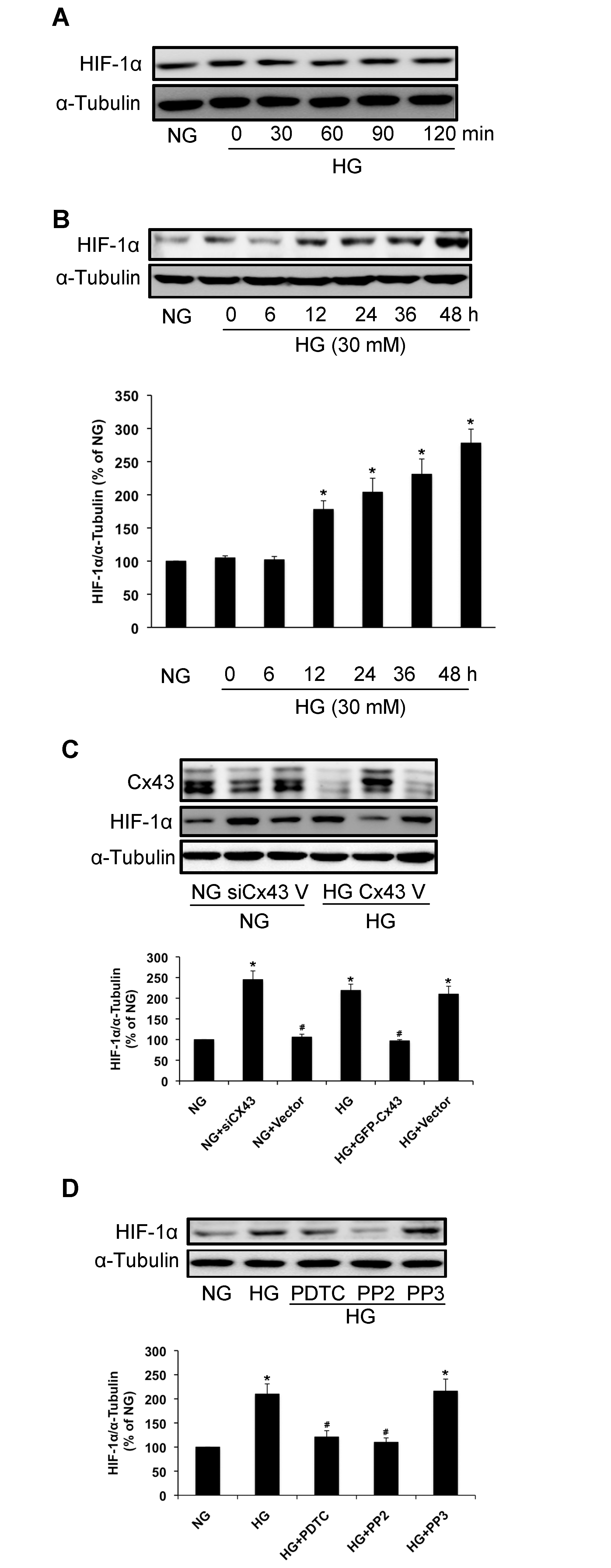

Supplement: Additional file 2: Figure S2 — HIF-1α is regulated in the GMCs by high glucose or low levels of Cx43. (A and B) GMCs were treated with high glucose for the indicated time, then proteins were extracted for analysis of HIF-1α by immunoblotting. (C) GMCs were transfected with Cx43-siRNA in normal glucose (5.5 mM) or GFP-Cx43 in high glucose (30 mM). After 48 h, proteins were extracted for analysis of Cx43 and HIF-1α by immunoblotting. (D) GMCs cultured in high glucose were co-incubated with PP2 or PP3 (10 μM) or PDTC (100 μM). After 48 h, proteins were extracted for analysis of HIF-1α by immunoblotting. α-Tubulin was used as a loading control. *P<0.05 vs. normal glucose-treated group. #P<0.05 vs. high glucose-treated group. [file 1478-811X-11-38-S2.tiff]
